# Risk factors, endoscopic findings, and treatments in upper gastrointestinal bezoars: multi-center experience in Iran 

**Published:** 2021

**Authors:** Gholamreza Hemmasi, Elahe Zanganeh, Seyed Ashkan Hosseini, Mehdi Alimadadi, Anahita Ahmadi, Shahin Hajiebrahimi, Mohammadreza Seyyedmajidi

**Affiliations:** 1 *Iran University of Medical Sciences, Tehran, Iran*; 2 *Golestan Research Center of Gastroenterology and Hepatology (GRCGH), Golestan University of Medical Sciences, Gorgan, Iran*; 3 *Mashhad University of Medical Sciences, Mashhad, Iran*

**Keywords:** Bezoar, Risk factors, Endoscopic intervention

## Abstract

**Aim:**

The current study aimed to investigate the risk factors, endoscopic findings, and treatments of upper gastrointestinal bezoars.

**Background:**

Bezoars are compact masses formed by the accumulation of dietary fiber, undigested food, hair, or medications. The majority of bezoars are asymptomatic, but they may cause serious symptoms or even life-threatening events such as bleeding, obstruction, or perforation

**Methods:**

This retrospective study was conducted in three gastroenterology clinics between January 2016 and December 2019. Bezoars were detected in 109 of 15,830 endoscopy records (0.68%).

**Results:**

A total of 103 patients (52.4% male) were enrolled in this study. Mean patient age was 60.5±11.3 years. The most frequent risk factors were history of gastric surgery (25.2%), diabetes mellitus (21.3%), hypothyroidism (15.5%), trichophagia (5.8%), and anxiety disorders (2.9%), respectively. The most common endoscopic findings were peptic ulcers (34.9%), erosive gastritis/duodenitis (12.6%), and reflux esophagitis (10.6%). While bezoars were most commonly observed in the stomach (84.4%), the majority of them were phytobezoars (92.2%). The mean number of endoscopic interventions for each patient was 1.5 (range, 1-4). Endoscopy was successful in removing bezoars in 85.4%.

**Conclusion:**

The synergistic effect of multiple factors for a long time, such as gastrointestinal surgery, diabetes mellitus or psychiatric disorders, may lead to bezoar formation. These risk factors should be avoided or treated in order to prevent bezoar formation and subsequent life-threatening complications.

## Introduction

 Bezoars are compact masses formed by the accumulation of materials, including dietary fiber, undigested food, hair, or medications. The word bezoar originates from the Persian word “padzahr”, meaning a counter-poison or antidote applied to describe a greenish, hard concretion in the stomach. In 1854, Richard Quain, an Irish anatomist of the University of London, reported a mass in the stomach found on autopsy that he called “bezoar” (1). Upper gastrointestinal (GI) bezoars are most commonly observed in the stomach. They may also be discovered in the esophagus, duodenum, and other segments of the bowel (2-4). In adolescents and young ladies, gastric bezoars are associated with a history of pica or psychiatric disorders; in adults, however, they are accompanied by gastroparesis, anatomical abnormalities, and previous gastric surgeries that have reduced gastric motility and delayed stomach emptying. Hypothetically, partially digested or undigested materials and gastric mucus can be a source of a gastric bezoar (5).

Bezoars are categorized according to the materials that form them: (a) Phytobezoars: composed of indigestible fruits or vegetables; (b) Trichobezoars: composed of hair; (c) Lactobezoars: composed of milk products; (d) Pharmacobezoars: composed of tablets and medications (5). Other substances may also contribute to their formation, such as metals, ceramics, fungi (candida), plastic, parasites (ascaris), and paper (1). The majority of patients are asymptomatic, though some patients may present with nausea, vomiting, epigastric pain, early satiety, bloating, and weight loss (6). Patients with esophageal bezoars may present with dysphagia, retrosternal pain, and gastroesophageal reflux (7). Bezoars have clinical importance because of their complications such as bleeding, obstruction, perforation, and fistulization (8).

Bezoars are diagnosed either endoscopically or radiologically. Contrast-enhanced computed tomography (CT) scanning is the radiological method of choice. Phytobezoars, the most common type, are visualized as round or ovoid mass-like defects filled with air-bubbles. Trichobezoars often have a lamellated appearance. A CT scan also enables the detection of multiple bezoars and the exclusion of other causes of obstruction and permits accurate preoperative planning (9). The gold standard is direct visualization with upper GI endoscopy for both diagnostic and therapeutic purposes. Bezoars normally appear in the gastric fundus in various colors (green, black, beige, or other, according to composition). They may be multiple or impacted in the esophagus or duodenum (10). Gastric bezoars can be managed with endoscopy or surgery.

This study was conducted in three tertiary centers in Iran (Academic hospitals of Iran, Golestan and Mashhad University of Medical Sciences). Current data concerning the risk factors, diagnosis, and therapy of upper GI bezoars was investigated. 

## Methods

This retrospective survey was conducted by examining the files of patients who had undergone upper GI endoscopy in three gastroenterology clinics between January 2016 and December 2019. Patients in whom bezoar was detected in their endoscopic examination were enrolled. In 109 of 15,830 endoscopy records (0.68%), bezoars were detected. Six of these patients were excluded because of imperfect clinical data, and the remaining 103 patients were included. The demographic characteristics, risk factors, endoscopic findings, type, location, and size of bezoars, number of endoscopic sessions, endoscopic success rate, and causes of treatment failure were recorded. Polypectomy snare, basket, mechanical lithotripter, and/or over-tube were used to fragment the bezoars. As necessary, 200 to 500 mL of carbonated beverage irrigations were administered. Successful endoscopic treatment was defined as complete fragmentation or extraction of bezoars. Patients with treatment failure were referred to surgery.

All the data was analyzed using Statistical Package for Social Sciences Ver.20 (SPSS, IBM Corp., Armonk, NY, USA). The values were expressed as mean ± standard deviation (SD) for continuous variables and percentages for categorical variables. The normality of distribution of data was assessed using the Kolmogorov-Smirnov test. 

## Results

A total of 103 patients, of whom 54 (52.4%) were male, were enrolled in this study. The mean age of the patients was 60.5±11.3 years, and 76 patients had the following risk factors: history of GI surgery in 26 patients (25.2%), including one patient with bariatric surgery; diabetes mellitus in 22 patients (21.3%); hypothyroidism in 16 patients (15.5%); trichophagia in 6 patients (5.8%);, anxiety disorders in 3 patients (2.9%); anorexia nervosa in one patient; autism in one patient; and scleroderma in one patient. 

The most common endoscopic findings were gastric ulcer and/or duodenal ulcer in 36 patients (34.9%), erosive gastritis/duodenitis in 13 patients (12.6%), and reflux esophagitis in 11 patients (10.6%). Less common findings were duodenal diverticula in two patients, esophageal stenosis in one patient with scleroderma, and the presence of a metal foreign body in the stomach of one patient. The risk factors and endoscopic findings of the patients are summarized in Table 1. 

**Table 1:** Patients’ risk factors and endoscopic findings

Risk factors	N (%)
Gastrointestinal surgery Stomach / Duodenum Bariatric surgery Esophagus (Fundoplication) Diabetes mellitus Hypothyroidism Trichophagia Anxiety disorders Anorexia nervosa Autism Scleroderma	26 (25.2) 24 (21.3) 1 (0.9) 1 (0.9) 22 (21.3) 16 (15.5) 6 (5.8) 3 (2.9) 1 (0.9) 1 (0.9) 1 (0.9)
Endoscopic findings	
Gastric ulcer and/or duodenal ulcer Erosive gastritis/duodenitis Reflux esophagitis Duodenal diverticula Esophageal stenosis due to scleroderma Metal foreign body in the stomach	36 (34.9) 13 (12.6) 11 (10.6) 2 (1.9) 1 (0.9) 1 (0.9)

As shown in Table 2, bezoars were most commonly observed in the stomach in 87 patients (84.4%). They were also detected in the duodenum in 13 patients and esophagus in 2 patients. One patient had bezoars in both the stomach and the duodenum. One of two patients with bezoars in the esophagus had a history of fundoplication surgery and another one had scleroderma. Moreover, 95 patients (92.2%) had phytobezoar, 6 patients had trichobezoar (all of whom had a history of trichophagia), one patient had pharmacobezoar, and one patient had a metal foreign body with undigested food.

**Table 2:** Bezoar locations and types

Bezoar locations	N (%)
Stomach Duodenum Esophagus Stomach–duodenum	87 (84.4) 13 (12.6) 2 (1.9) 1 (0.9)
Bezoar type	
Phytobezoar Trichobezoar Pharmacobezoar Metal	95 (92.2) 6 (5.8) 1 (0.9) 1 (0.9)

The mean measurement of the long side of the bezoars was 5.5±2.3 cm (range, 2 to 10 cm in diameter). The mean number of endoscopic interventions for each patient was 1.5 (range, 1-4). Bezoars were successfully removed with endoscopic interventions in 88 (85.4%) patients. In 4 of 6 patients with trichobezoar, endoscopic intervention attempts failed to fragment the trichobezoar. Another 11 patients had phytobezoars measuring more than 7 cm in diameter and underwent 3 or 4 endoscopic intervention sessions with no success (Table 3). These patients were referred for surgery.

**Table 3:** Number of endoscopic sessions and endoscopic treatment failures

Endoscopic session numbers	N (%)
1 2 3 4	66 (64.1) 26 (25.2) 8 (7.8) 3 (2.9)
Endoscopic treatment failure	15 (14.6)
Trichobezoar resistant to fragment with endoscopic intervention Phytobezoars more than 7 cm in diameter	4 of 6 (66.7) 11 of 95 (11.6)

## Discussion

With human aging, bezoars become an increasingly recognized disorder. Past GI surgery and dysmotility, medications, and diabetes mellitus, which increasingly affect people of older ages, are the predominant predisposing factors for bezoar types (1). In the current study, 15,830 endoscopy records were examined and 109 (0.68%) patients with bezoar were detected. A total of 103 patients with upper GI bezoars diagnosed within the last 4 years were studied; 52.4% of them were male, and mean patient age was 60.5±11.3 years. In other studies, bezoar rate and mean patient age were reported as between 0.06% to 0.09% and 58-63 years, respectively (2, 11). These results indicate that bezoars occur in patients who are relatively older. 

The most common risk factor in this study was history of GI surgery. In the literature (2, 12-14), the rate of past GI surgery differed from 23% to 57% compared to 25.2% in the current study. The fact that one of two patients with bezoar in the esophagus had a history of fundoplication surgery and another one had scleroderma indicates that motility disorders are an important risk factor for esophageal bezoars. In the current study, 21.3% of patients had diabetes mellitus and 15.5% had hypothyroidism. In these patients, a decrease in GI motility precipitated bezoar formation. Furthermore, gastric acid secretion may be reduced, and this condition may precipitate bezoar formation (15). Psychiatric disorders, including trichophagia, anxiety disorders, anorexia nervosa, and autism, were other important risk factors for bezoar formation in the current study. The association of trichophagia and bezoar (Rapunzel syndrome) has been reported (16). Several reports have also suggested an association between generalized anxiety disorder and bezoar formation (17).

The most common endoscopic findings were peptic ulcers, erosion, and reflux esophagitis. The main reason for these results may be the fact that patients might have a prolonged exposure to gastric acid and decreased motility. Another possible mechanism may be the pressure effect of the bezoar (2). The most common location of bezoars in this study was the stomach. Case series in the literature had similar findings (5). There are rare reports of giant bezoars extending from the stomach to the duodenum or in multiple locations (18), as was seen in one patient in the current study. The most common bezoar type in the literature, as in the current study, was phytobezoar (19).

Gastric bezoars can be managed with medicine, endoscopy, or surgery: (a) Enzymatic treatment include carbonated beverage irrigations, gastroprokinetic agents, and enzyme cellulose; (b) Endoscopic management as the main treatment includes alligator forceps, lithotripters, snares of polypectomy, and laser-ignited lithotripsy; (c) Surgical management is the best treatment for bigger bezoars (5). The success rate of endoscopic intervention in the treatment of bezoar was 85.4% in this study, which is similar to several other studies (20). The treatment success rate in the current study might have been influenced by the fact that dissolvers like cola were used optionally. The main reason for failure with endoscopic treatment was the size of bezoars (more than 7 cm in diameter). In addition, endoscopic intervention attempts failed to fragment the trichobezoar in 4 of 6 such patients. A literature search showed that surgical treatment is preferred over endoscopic treatment in patients with trichobezoar (21). Kajal et al. (22) suggested that the main treatment option for trichobezoar is surgery because of late presentation in these patients, and the prevention of recurrence requires concomitant treatment of psychiatric disorders such as trichophagia. Although endoscopic treatment is prolonged and repeated sessions may be required, this management is still the initial option of choice for all types of bezoar because of the complications of surgery (5). 

In conclusion, bezoars most frequently occur in patients with risk factors including past GI surgery, anatomic abnormalities, weakened gastric motility, psychiatric disorders, or coexisting medical conditions. The synergistic effect of multiple factors for a long time may lead to bezoar formation. Future research should investigate the formation mechanism of bezoars in order to prevent their formation and subsequent life-threatening complications.
